# A method for EMCCD multiplication gain measurement with comprehensive correction

**DOI:** 10.1038/s41598-021-85511-z

**Published:** 2021-03-15

**Authors:** Li Qiao, Mingfu Wang, Zheng Jin

**Affiliations:** Institute of Optics and Electronics, Chinese Academy of Sciences, Chengdu, 610209 Sichuan China

**Keywords:** Computational science, Computer science, Scientific data, Statistics

## Abstract

In order to improve the image quality, it is imperative to conduct the non-uniformity correction of EMCCD, for which the measurement accuracy of the internal electron multiplication gain of each channel is a prerequisite within multi-channel output EMCCD. It is known that the smaller the image standard deviation of each channel, the better the image uniformity, and the closer the calculated multiplier gain is to the real value. In order to minimize the influence of non-uniformity of background between pixels and light response existing in traditional measurement, a comprehensively modified EMCCD multiplication gain measurement is proposed after the working principle of EMCCD is described. The output images of the camera working in the normal CCD mode and EMCCD mode are corrected comprehensively through this method. The experimental results show that after the comprehensive correction, the standard deviation of the output image of each channel within the camera decreases to about one third of the original when the camera works in the normal CCD mode, while it decreases to about one fifth of the original when the camera works in the EMCCD mode, the signal stability is significantly improved, and the measured multiplier gain of each channel is closer to the true value of the detector, which proves the effectiveness of the proposed method.

## Introduction

EMCCD is known as electron multiplying charge coupled device. Compared with the traditional CCD, EMCCD adds an electron multiplier register (EMR) between the readout register and the readout amplifier^1–3^. The EMR can amplify the weak electrical signal exponentially, so as to achieve the purpose of multiplication. This process can greatly improve the detection sensitivity of EMCCD, making it one of the most widely used high-end detectors in the field of low-light imaging fields.

Non-uniformity correction of EMCCD is the key to improve image quality. There are many factors affecting the non-uniformity of common CCD, such as the non-uniformity of background and light response, dark current, device aging, etc. The non-uniformity of EMCCD includes not only the non-uniformity component of common CCD, but also the non-uniformity caused by multiplication gain: (1) non-uniformity between different channels. The multi-tap output structure adopted by EMCCD chip can improve the maximum output frame rate of the detector, which, simultaneously, will also introduce the non-uniformity caused by the difference of real multiplication gain between image channels; (2) non-uniformity between pixels within channel. The multiplication of photo-generated charge in EMCCD's electron multiplier register is a random process, which brings in discrepancies of multiplication gain between pixels and introduces non-uniformity. When the multiplication gain increases, the introduced non-uniformity often increases as well, which will even be the main component of the non-uniformity of EMCCD under the extremely weak light imaging condition. In the process of true multiplier gain measurement, the effective signal is both affected by the non-uniformity of background and optical response, which leads to the difference between the actual measurement and the true multiplier gain. At present, the research on non-uniformity correction mainly focuses on infrared imaging and common CCD imaging^4–11^. The related scientific research of EMCCD mainly concentrates on the application of weak light imaging in astronomical observation^12–19^, biomedical^20–26^ and other fields. There are few literatures on the non-uniformity characteristics of EMCCD imaging and its correction. The comprehensive correction method proposed in this paper can greatly reduce the influence of background non-uniformity and optical response non-uniformity on the effective signal for calculating the multiplier gain. After comprehensive correction, the standard deviation of the output image of the 8-channel EMCCD detector CCD220 from e2v company is significantly decreased, and the image quality is significantly improved, the real multiplier gain of each channel of EMCCD can be obtained more accurately from the comprehensively corrected image.

## Mechanism of EMCCD

**Figure 1:**
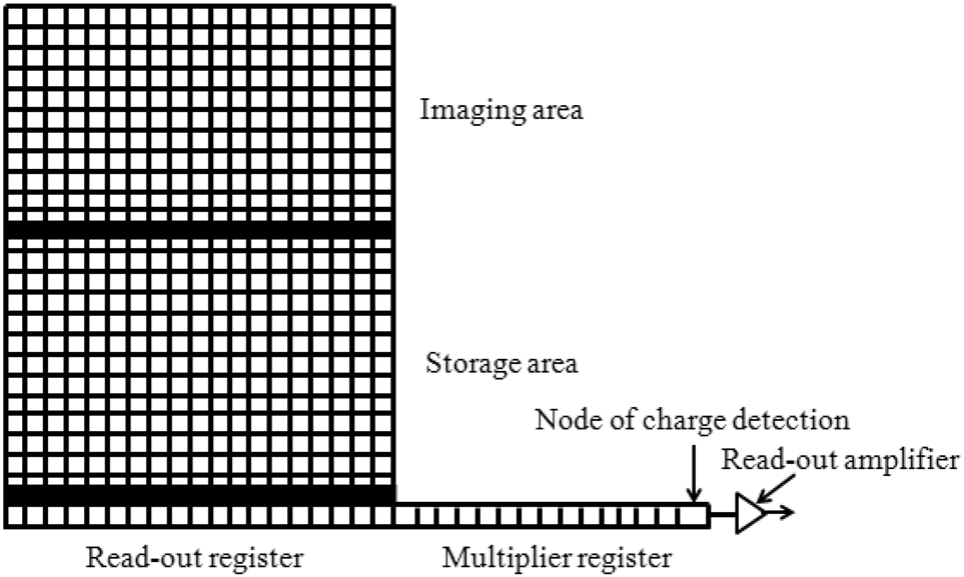
Schematic diagram of EMCCD structure.

The internal structure of EMCCD is mainly composed of imaging, store, read-out register, multiplier register, readout amplifier and back-end circuit^2^. Photo generated charge is formed when the imaging area is irradiated by the external light, the transfer process is shown in Fig. 1. Firstly, the photo generated charge will be transferred to the storage area continuously within the specified exposure time and then to the readout register sequentially, where the photo generated charge will be sent to the multiplication register for exponential multiplication and then to the readout amplifier and the back-end circuit for processing, and finally the image will be output.

The electron multiplier register is a multi-stage multiplier register. The real multiplication gain of photo-generated charge in the electron multiplier register is shown in Eq. (1)^27^:

1 $$G={\left(1+r\right)}^{N}$$

In this equation, *G* is the number of electrons output after a single electron is amplified by the multiplier register, that is, the true multiplier gain (no unit). *N* is the number of electron multiplication units in the multiplier register (no unit), which is 520 for CCD220. *r* is the additional multiplication coefficient (*r* ≥ 0, no unit). When *r* = 0, the camera works in normal CCD mode; when *r* > 0, the camera works in EMCCD mode. The larger the gain voltage of the multiplier register, the greater *r* and *G* and vice versa.

## Measurement of multiplication gain based on comprehensive correction

### Principle of correction

The true multiplier gain of the multiplier register is the ratio of the signal after multiplication to the one before multiplication. The calculation formula is as follows:

2 $$G=\frac{S(gain)}{S(no\;gain)}=\frac{S\left(bright,gain\right)-S(dark,gain)}{S\left(bright,no\;gain\right)-S(dark,no\;gain)}$$

In this equation, *G* is the true multiplication gain of the multiplier register; $S\left(bright,gain\right)$ is the bright field signal after multiplication; $S(dark,gain)$ is the dark field signal after multiplication; $S\left(bright,no\;gain\right)$ is the bright field signal without multiplication; $S(dark,no\;gain)$ is the dark field signal without multiplication; $S(no\;gain)$ is the original input signal of the multiplier register; $S(gain)$ is the output signal after multiplication. The accuracy of *G* is directly affected by $S(gain)$ and $S(no\;gain)$. Therefore, it is necessary to comprehensively correct the input and output signal of the multiplier registers in advance according to the existing non-uniformity characteristics of the background and the light response between pixels. The corrected multiplier gain is calculated as follows:

3 $${G}^{^{\prime}}=\frac{{S}^{^{\prime}}(gain)}{{S}^{^{\prime}}(no\;gain)}=\frac{f\left[S\left(bright,gain\right)\right]-f\left[S(dark,gain)\right]}{f\left[S\left(bright,no\;gain\right)\right]-f\left[S(dark,no\;gain)\right]}$$

${G}^{^{\prime}}$ is real multiplier gain calculated after comprehensive correction; $f$ is the comprehensive correction function of EMCCD output image.

### Determination of comprehensive correction function

#### Non-uniformity composition

The dark field image is the output of camera under condition of no light (no photoelectric conversion), while the bright field image is the one under the condition of light (photoelectric conversion). The background image is the dark field image when the camera is set to the minimum exposure time. The light input of the camera is adjusted by controlling the exposure time when the brightness of the light is kept constant. The gray value of bright field image is the sum of those of background image and light response signal, as shown in formula (4)

4 $${image}_{dark0}(i,j)+k(i,j)*A*{t}_{exp}+b(i,j)={image}_{bright}(i,j)$$

In this equation, $i,j$ is the *i*th row and *j*th column of the pixel in the image; $A$ (unit: photons/s) is the coefficient related to the brightness of the light source; ${t}_{exp}$ is the exposure time; ${image}_{dark0}$ is the background image; $k$, $b$ is the slope and intercept of the light response curve of a single pixel respectively (hereinafter referred to as the light response coefficient); $photons$ is the light input of the camera. The difference of gray value between pixels within background image incurs the background non-uniformity of camera, while the difference of light response coefficient $k$ and $b$ between pixels brings in the non-uniformity of light response. Therefore, the background non-uniformity and light response non-uniformity are introduced into the bright field image. The light response signal is the difference between the bright field image and the background image, which effectively removes the non-uniformity of the background image and only retains the non-uniformity of the light response. By determining the corresponding background gray value and light response coefficient of each pixel, the output bright field image can be comprehensively corrected.

#### Determination of correction coefficient

The mean image can effectively eliminate the influence of random noise, and its calculation formula is as follows:

5 $${image}_{ave}(i,j)=\frac{\sum_{m=1}^{n}{image}_{m}(i,j)}{n}$$

In this equation, ${image}_{m}$ is a single image; n is the number of collected images; ${image}_{ave}$ is the calculated mean image.

The background image ${image}_{dark0}$ is obtained by fitting the camera under the dark condition, setting the exposure time to the minimum, collecting various dark field images, and then calculating the mean image according to formula (5).

The brightness of the light source is remained unchanged and multiple bright field images are captured at *m* exposure times ${t}_{exp}$, then the bright field mean image is calculated. By subtracting *m* background mean images from bright field mean images, the light response signal of each pixel ${image}_{diff}$ can be obtained. Finally, the light response coefficient of a single pixel can be obtained by linearly fitting as shown in formula (6)

6 $${image}_{diff}\left(i,j\right)= k\left(i,j\right)*A*{t}_{exp}+b(i,j)$$

In this equation, $k*A$ and $b$ are the fitted light response coefficients.

The following can be drawn from formula (4):

7 $${t}_{exp}=\frac{{{image}_{bright}\left(i,j\right)-image}_{dark0}(i,j)-b(i,j)}{k\left(i,j\right)*A}$$

In this equation, ${image}_{bright}$ is the bright field image to be corrected, ${image}_{dark0}$ is the background image (equivalent to dark field image).

#### Comprehensive correction

The mean value of the whole image is shown in formula (8):

8 $${ave}_{image}=\frac{\sum_{i=1}^{row}\sum_{j=1}^{col}image(i,j)}{row*col}$$

In this equation, $row,col$ are the total number of rows and columns respectively, $image$ is the single image; ${ave}_{image}$ is the mean value of the whole image.

According to formula (8), the mean values of the background image ${image}_{dark0}\left(i,j\right)$, light response coefficients $k\left(i,j\right)*A$ and $b\left(i,j\right)$ are calculated to be ${image}_{dark0\_ave}$, ${k}_{ave}*A$, ${b}_{ave}$, respectively.

The bright field image after comprehensive correction can be calculated from its composition as follows:

9 $${image}_{modify}\left(i,j\right)={image}_{dark0\_ave}+{k}_{ave}*A*{t}_{exp}(i,j)+{b}_{ave}$$

Apply formula (7) into (9), the following results can be obtained:

10 $${image}_{modify}\left(i,j\right)={k}_{total}\left(i,j\right)*{image}_{bright}\left(i,j\right)+{b}_{total}\left(i,j\right)$$

11 $${k}_{total}\left(i,j\right)=\frac{{k}_{ave}*A}{k\left(i,j\right)*A}$$

12 $${b}_{total}\left(i,j\right)={image}_{dark0\_ave}+{b}_{ave}-\frac{{k}_{ave}*A*\left[{image}_{dark0}\left(i,j\right)+b\left(i,j\right)\right]}{k\left(i,j\right)*A}$$

In formulas (10)–(12), ${image}_{ave\_dark0}$ is the mean value of the whole image of ${image}_{dark0}$; ${image}_{modify}$ is the image after comprehensive correction.

The comprehensive correction function corresponding to function *f* in formula (3) is shown in Eqs. (13–15):

13 $$Y\left(i,j\right)={k}_{total}\left(i,j\right)*X\left(i,j\right)+{b}_{total}\left(i,j\right)$$

14 $${k}_{total}\left(i,j\right)=\frac{{k}_{ave}*A}{k\left(i,j\right)*A}$$

15 $${b}_{total}\left(i,j\right)={image}_{dark0\_ave}+{b}_{ave}-\frac{{k}_{ave}*A*\left[{image}_{dark0}\left(i,j\right)+b\left(i,j\right)\right]}{k\left(i,j\right)*A}$$

In this equation, $X$ is the original image to be corrected; $Y$ is the image after comprehensive correction.

### Measurement procedure

**Figure 2:**
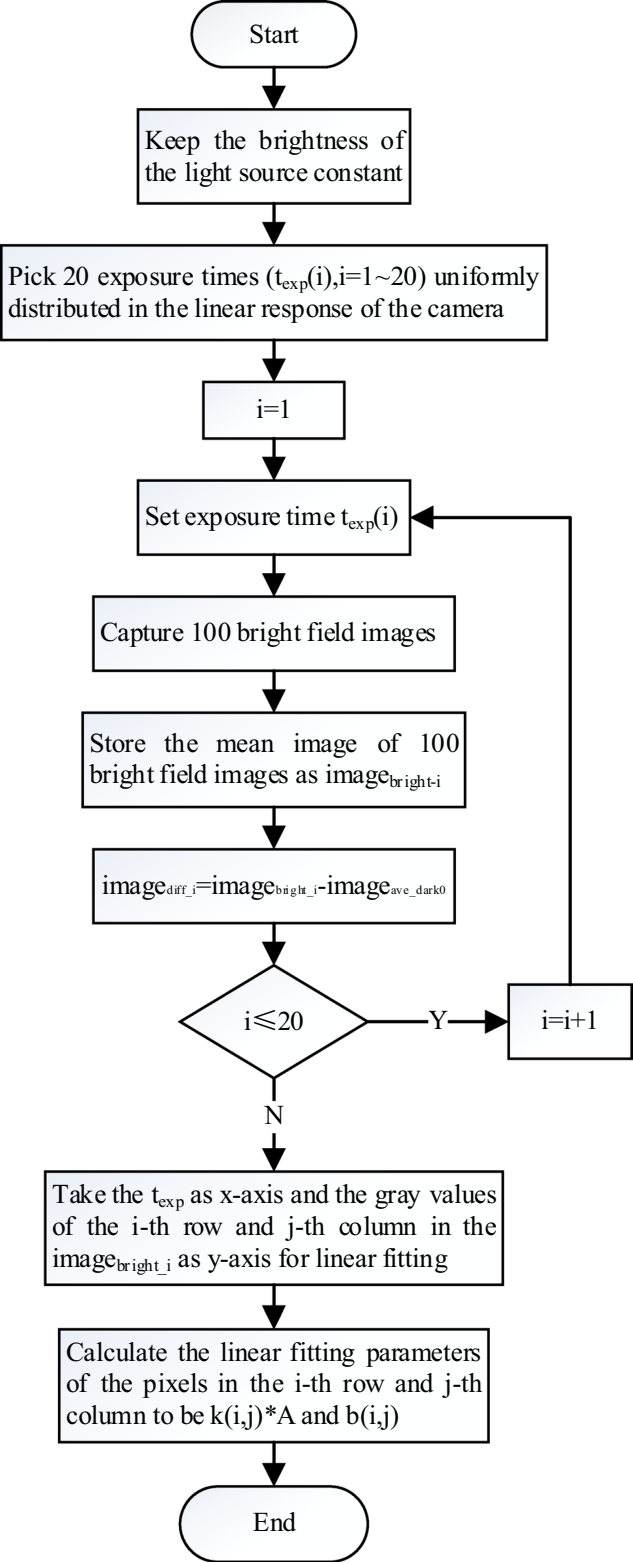
Calculation flow chart of light response coefficient of single pixel.

According to formulas (14) and (15), it is necessary to determine six factors in Table 1 for the comprehensive correction of the original image of the camera. The more the number of collected images, the smaller the random error of the mean image. Therefore, 100 background images were collected to calculate ${image}_{dark0}$ in this paper according to formula 5, then ${image}_{ave\_dark0}$ can be obtained by substituting ${image}_{dark0}$ into formula (8). The process of obtaining the light response coefficients of the pixels in the *i*th row and *j*th column is shown in Fig. 2. As the light response coefficients are obtained from linear fitting, the more sampling points, the more accurate the result is. In this paper, 20 exposure time points are used for linear fitting. The slope $k*A$ and intercept $b$ of the light response curve can be calculated after the operation for all pixels as shown in Fig. 2, the mean value of slope ${k}_{ave}*A$ and intercept ${b}_{ave}$ can be obtained from formula (8).

**Table 1:** Correction factors to be determined.

Number	Factor	Symbol
1	Background image	${image}_{dark0}$
2	Average background image	${image}_{dark0\_ave}$
3	Slope of light response curve	$k*A$
4	Average slope of light response curve	${k}_{ave}*A$
5	Intercept of light response curve	$b$
6	Average intercept of light response curve	${b}_{ave}$

After the parameters in Table 1 are calculated, the comprehensive correction coefficient ${k}_{total}$, ${b}_{total}$ are obtained by substituting them into formulas (14) and (15). Finally, ${k}_{total}$, ${b}_{total}$ and the original output image are substituted into formula (13) as $X\left(i,j\right)$ to get the camera output image with comprehensive correction. By sending the light field image and dark field image corrected into formula 2, the final true multiplier gain value with comprehensive correction can be calculated.

## Experimental results

The CCD220 chip of British e2v company possesses sub-electron readout noise capability and excellent spectral response characteristics, which is a typical high frame rate EMCCD chip used in adaptive optics. CCD220 chip adopts 8-channel parallel output mode, which can improve the maximum output frame rate of the camera. The resolution of the final output image is 240 × 240, and it is composed of eight channels. The non-uniformity of the multiplication gain between channels will directly incur the non-uniformity of the original output image, which produces eight distinct regions. In this paper, CCD220 chip is taken as an example to compare the calculation results of the traditional method with the new proposed one.

### Experimental equipment and parameters

**Figure 3:**
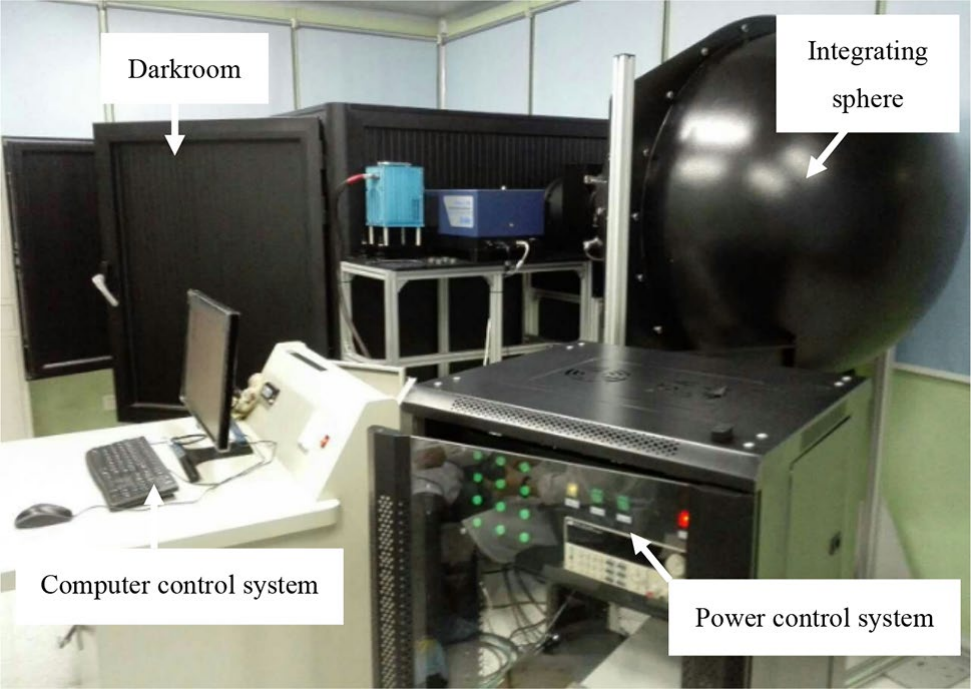
Integrated test platform of EMCCD.

Figure 3 shows the hardware equipment of this experiment, which is composed of integrating sphere, darkroom, power control system and computer control system. The camera is installed on a special bracket in the darkroom, and the CCD220 is facing the center of the integrating sphere light outlet. The distance between the target surface of the detector and the light outlet of the integrating sphere is about 1.2 m. The integrating sphere has four levels of transmittance of 100%, 10%, 1% and 0.1%, which can provide the normal light environment for the camera when it works in the non-multiplication gain mode and the weak light environment when it works in the high multiplication gain mode.

According to the chip manual, CCD220 works in the normal CCD mode when the gain voltage is 17 V, and the true multiplier gain *G* is 1, which raises exponentially with the increase of the gain voltage. Therefore, the output image of the camera was measured in experiment when the gain voltage was 17 V (with the multiplier register off) and 34 V (with the multiplier register on). The relevant test parameters are shown in Table 2.

**Table 2:** EMCCD working parameters.

Current of integrating sphere (A)	0.8
Transmittance of integrating sphere	100%
Exposure time (ms)	0.5
Voltage of gain register (V)	17, 34

### Experimental result

#### Comparison of images

**Figure 4:**
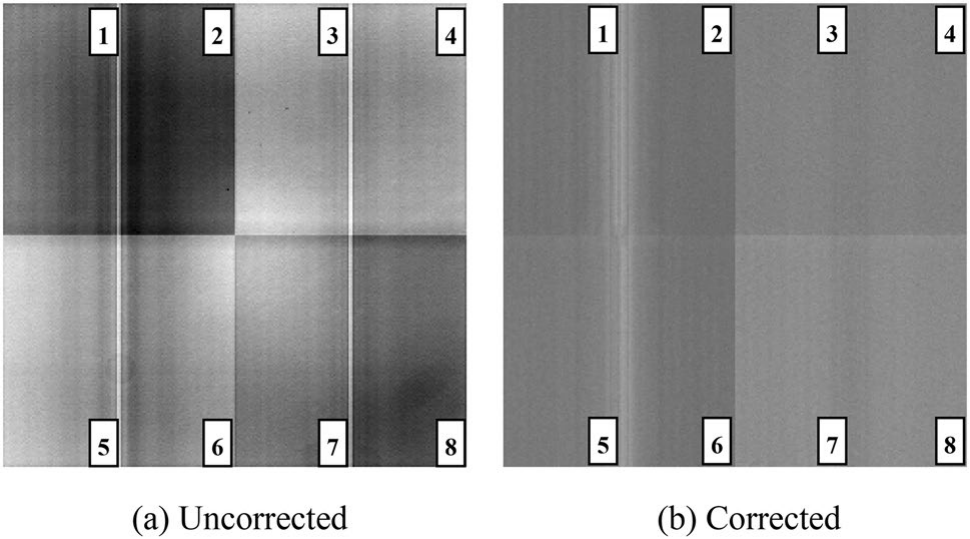
Comparison of CCD220 output image before and after correction when the gain register voltage is 17 V.

**Figure 5:**
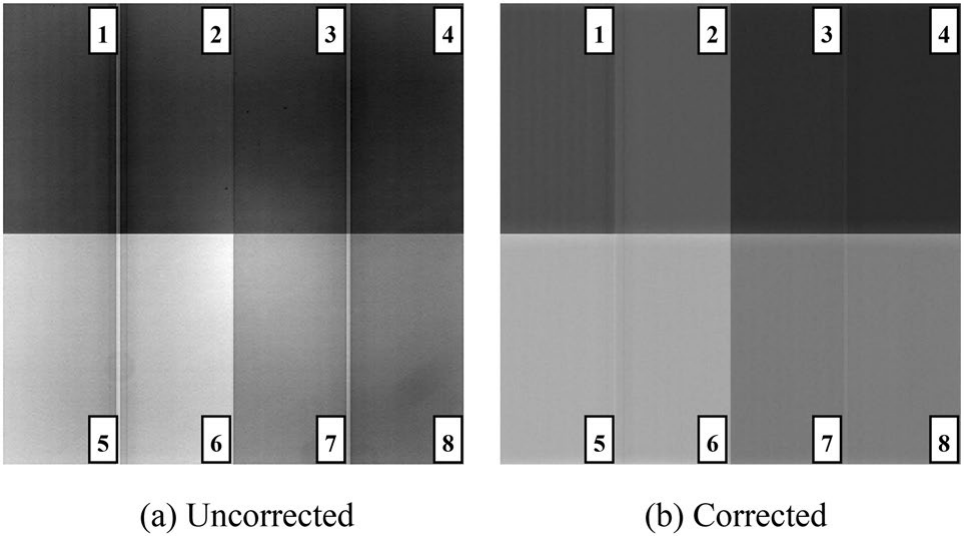
Comparison of CCD220 output image before and after correction when the gain register voltage is 34 V.

Figures 4 and 5 show the original output image and the one after the comprehensive correction obtained by CCD220 detector when the gain voltage is 17 V and 34 V, respectively.

The output image of CCD220 consists of 8 channels, namely eight rectangular regions in the single image as shown in Figs. 4 and 5. The four regions in the upper half are named channel 1–4, similarly the four in the lower half are named channel 5–8. It can be seen from the above figure that the uniformity of the output image in a single channel has been significantly improved after comprehensive correction, the most intuitive difference is that there is a noticeable bright spot in the center of the whole image before correction but it nearly dies away after correction.

#### Statistical comparison

The internal standard deviation of single channel image is calculated as follows:

16 $${std}_{image}=\sqrt{\frac{\sum_{i=1}^{row}\sum_{j=1}^{col}{(image\left(i,j\right)-{image}_{ave})}^{2}}{row*col-1}}$$

In this equation, ${std}_{image}$ is the standard deviation of the single channel image.

In this experiment, the integrated test platform of EMCCD adopts the integrating sphere as the system light source, with the brightness uniformity of the light outlet more than 97% and the brightness stability less than 0.05% within 30 min. Ideally, the standard deviation of the effective signal for calculating the multiplier gain should be 0 DN, namely the smaller the standard deviation of the effective gain signal is, the more stable the signal is, and the closer the calculated multiplier gain is to the true value. Tables 3 and 4 show the 8-channel standard deviation of the original image and the one after comprehensive correction, respectively, when CCD220 gain register voltage is set to be 17 V and 34 V.

**Table 3:** Standard deviation of original 8-channel image.

Gain voltage (V)	Standard deviation (DN)								
	Channel 1	Channel 2	Channel 3	Channel 4	Channel 5	Channel 6	Channel 7	Channel 8	Total image
17	16.59	15.50	15.72	12.10	16.15	18.21	20.77	15.37	16.30
34	71.35	69.67	66.63	50.25	74.95	85.28	89.81	62.93	71.36

**Table 4:** Standard deviation of 8-channel image after comprehensive correction.

Gain voltage (V)	Standard deviation (DN)								
	Channel 1	Channel 2	Channel 3	Channel 4	Channel 5	Channel 6	Channel 7	Channel 8	Total image
17	6.50	8.11	2.35	2.33	4.16	8.09	2.82	2.63	4.62
34	13.07	13.61	13.18	12.83	14.48	14.65	15.20	14.97	14.00

**Figure 6:**
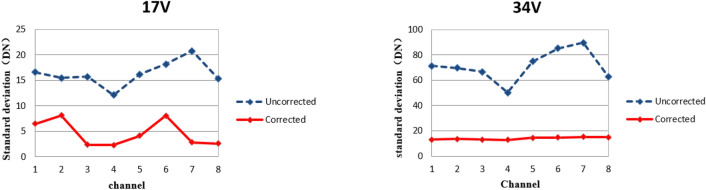
Standard deviation of CCD220 8-channel image before and after correction under different gain voltages.

Figure 6 shows the trend of standard deviation of 8-channel effective signal when the gain voltage of CCD220 is 17 V and 34 V, respectively. The blue dotted line represents the uncorrected image while the red solid line represents the comprehensively corrected one. It can be seen from the figure that: (a) when the gain voltage is 34 V, the standard deviation is larger than that when the gain voltage is 17 V. The larger the gain voltage, the larger the real multiplier gain of the multiplier register. When the effective signal of the image is amplified by the multiplier gain, the noise of the image is also magnified. (b) Under the same gain voltage, the standard deviation of the output images of the eight channels is greatly reduced after correction, and the uniformity of the images in each channel is significantly improved.

As for CCD220, it is necessary to calculate the multiplication gain of each channel separately because of its special characteristic of 8-channel output at the same time. The non-uniformity of EMCCD output image mainly includes the non-uniformity of background and light response between pixels, and also the multiplication gain non-uniformity between channels. The proposed linear correction method in this paper can effectively reduce the background non-uniformity and the light response non-uniformity between pixels in a single channel. After the correction, the standard deviation of single channel output is greatly reduced, of which the uniformity is significantly improved, and the final calculated real multiplier gain of each channel is closer to the real value.

## Conclusion

In this paper, a method of EMCCD multiplication gain measurement with comprehensive correction is proposed. The background non-uniformity and light response non-uniformity of eight channels in the output image of CCD220 are investigated, and the comprehensive correction coefficient of the effective signal of multiplier gain in each channel is calculated. The accuracy of the real multiplier gain calculation in each channel of EMCCD can be improved through the comprehensive correction. It is found that after the correction method described in this paper the standard deviations of the effective signals of the eight channels are significantly reduced, the signal stability is significantly improved, and the measured multiplier gain of each channel is closer to the true value of the detector. This method not only provides a new way to measure the real multiplier gain of EMCCD, but also provides a theoretical basis for the non-uniformity correction of multi-channel EMCCD.
